# Olanzapine for the Prevention of Chemotherapy-Induced Nausea and Vomiting: A Comparative Study From Sudan

**DOI:** 10.1200/JGO.17.00216

**Published:** 2018-05-11

**Authors:** Alaa A.M. Osman, Moawia M.A. Elhassan, Blala H.A. AbdElrahim, Fatima H.A. Ahmed, Jihad B.H. Yousif, Malak A.M. Ahmed, Romisa H.A. Abdelhafeez, Umsalama M.Y. Ahmed

**Affiliations:** **All authors:** University of Gezira, Wad Madani, Sudan.

## Abstract

**Purpose:**

Chemotherapy-induced nausea and vomiting (CINV) is a distressing adverse effect. Neurokinin-1 receptor antagonist (NK1RA)–containing regimens are the standard regimens for CINV prophylaxis in patients with cancer receiving highly or moderately emetogenic chemotherapy (HEC or MEC). NK1RA agents are expensive and were not registered in Sudan. Recently, regimens containing olanzapine, the available and affordable medication in Sudan, were introduced as another option. This study aimed to compare the efficacy of an olanzapine-containing regimen with the antiemetic regimen that was currently used in our institute for CINV prophylaxis in HEC/MEC settings.

**Patients and Methods:**

The study prospectively compared an olanzapine-containing regimen (acute phase: olanzapine, ondansetron, dexamethasone; delayed phase: olanzapine, ondansetron) with an ondansetron/dexamethasone regimen (acute phase: ondansetron, dexamethasone; delayed phase: ondansetron) in adult patients with cancer receiving HEC or MEC. The study outcomes were complete response (CR; no emesis and no rescue medications) and nausea control (no nausea), which were assessed in the acute (0 to 24 hours), delayed (24 to 120 hours), and overall (0 to 120 hours) phases.

**Results:**

The study included 131 patients (olanzapine-containing: 50 patients; ondansetron/dexamethasone: 81 patients). CR and nausea control were higher in the olanzapine-containing than in the ondansetron/dexamethasone regimen (CR: acute phase, 86% *v* 71.6%; *P* = .086; delayed phase, 72% *v* 30.9%; *P *< .001; overall phase, 66% *v* 25.9%; *P *< .001; nausea control: acute phase, 86% *v* 74.1%; *P* = .127; delayed phase, 76% *v* 34.6%; *P *< .001; overall phase, 72% *v* 29.6%; *P *< .001). The major toxicity of olanzapine was grade 1 and 2 sedation or drowsiness (25 patients).

**Conclusion:**

An olanzapine-containing regimen has better efficacy for prevention of CINV in the HEC/MEC setting. Oncologists working in a limited-resource setting should be familiar with olanzapine-containing regimens, because NK1RA agents are not affordable and not easily available.

## INTRODUCTION

Chemotherapy-induced nausea and vomiting (CINV) remains a common and serious adverse effect. It is associated with a significant negative impact on quality of life and leads to decreased tolerability to subsequent chemotherapy cycles, changes in treatment plan, treatment failure, and increased use of health care resources.^1-7^

Many neurotransmitters and their receptors are involved in CINV, with serotonin (5-hydroxytryptamine 3 [5-HT3]), neurokinin-1 (NK-1), and dopamine being the most important ones. These receptors are the targets for many antiemetic agents used for the prevention of CINV, such as 5-HT3 receptor antagonist (5-HT3RA; eg, ondansetron), NK-1 receptor antagonist (NK1RA; eg, aprepitant), and dopamine receptor antagonists (eg, metoclopramide).^8,9^

The emetogenic potential of a particular chemotherapeutic agent is the main risk factor that increases incidence and severity of CINV, among others.^10,11^ Many guidelines for prevention of CINV have been developed by the American Society of Clinical Oncology (ASCO),^12,13^ the National Comprehensive Cancer Network (NCCN),^10^ the European Society of Medical Oncology, and the Multinational Association of Supportive Care in Cancer.^14^ For prophylaxis of CINV in patients receiving highly emetogenic chemotherapy (HEC) and moderately emetogenic chemotherapy (MEC), these guidelines recommend NK1RA-containing regimens (acute phase: a NK1RA, a 5-HT3RA, and dexamethasone; delayed phase: aprepitant and dexamethasone).^10,12-14^ Although NK1RA-containing regimens significantly improve the control of acute and delayed emesis in patients receiving HEC and MEC,^15^ these regimens are underused in resource-limited settings because of the unavailability and high cost of NK1RA agents.^16^

NCCN guideline version 2.2016 recommended an olanzapine-containing regimen (acute phase: olanzapine, palonosetron, and dexamethasone; delayed phase: olanzapine) as a less costly alternative for prevention of CINV in patients receiving HEC and MEC regimens.^17^ Olanzapine is an atypical antipsychotic with potential antiemetic effect gained through its antagonist activity at multiple receptor types involved in CINV, including serotonin (5-HT 2a, 5-HT 2c, 5-HT 3, and 5-HT6), dopamine (D1, D2, D3, and D4), muscarinic acetylcholine, and histamine (H1) receptors.^18^

To optimize prevention and management of CINV in patients with cancer in limited-income countries, regional guidelines should take into account limited health resources, clinical practice, and treatment availability and affordability. In Sudan, at public hospitals, patients with cancer receive free treatment, including chemotherapy and antiemetic drugs. The combination of ondansetron and dexamethasone was the most commonly used regimen for prophylaxis of CINV in HEC/MEC settings, because none of the NK1RA or other 5-HTRA agents were registered in Sudan, and this resulted in suboptimal control of CINV, especially in the setting of HEC.^19^ This study aimed to compare the efficacy of an olanzapine-containing regimen with the antiemetic regimen that was currently used in our institute for prophylaxis of CINV in patients receiving HEC or MEC regimens.

## PATIENTS AND METHODS

### Inclusion and Exclusion Criteria

This was a prospective comparative study conducted at the Department of Oncology at the National Cancer Institute, University of Gezira (NCI-UG) in central Sudan during the period from January 25 to March 15, 2017. This study included all adult patients with cancer (age > 16 years) who received any cycle of intravenous (IV) HEC or MEC regimens in an outpatient setting. Exclusion criteria were upper GI malignancies, pregnancy, patients receiving multiday IV chemotherapy regimens in the wards, and patients receiving herbal antiemetics.

### Ethical Approval

Ethical approval was obtained from the ethical committee of Gezira University–Faculty of Medicine. An informed verbal consent was obtained from each participant. The interviewer explained the aims and objectives of the study and its potential value.

### Study Design

In the NCI-UG, the registry office usually assigns all patients with cancer, on their first day of admission, to be treated in one of the four treatment units. In this prospective comparative study, patients treated in two treatment units were assigned to receive a regimen containing 10 mg/d of oral olanzapine daily from day 1 to 4 (HEC) or day 1 to 3 (MEC). Patients treated in the other two treatment units did not receive olanzapine (non–olanzapine-containing regimen). All patients in the two groups received the current NCI-UG prophylactic antiemetic regimen, consisting of ondansetron and dexamethasone in the following dosage and schedule: acute phase: ondansetron 8 to 16 mg IV and dexamethasone 8 to 16 mg IV on day 1, 30 to 60 minutes before chemotherapy; delayed phase: ondansetron 8 mg by mouth twice a day for 5 days.

The emetic risk of each chemotherapy agent was determined according to the classification of the NCCN guideline version 2.2016.^17^ Emetic risk of the chemotherapy regimen was determined based on the agent with the highest emetic risk.

### Study Outcomes

Emesis control or complete response (CR; defined as no nausea and no vomiting and no use of rescue antiemetic medication) was the primary outcome. Nausea control (defined as no nausea) was the secondary outcome. These outcomes were analyzed in the acute phase (0 to 24 hours after chemotherapy), delayed phase (24 to 120 hours after chemotherapy), and overall phase (0 to 120 hours after chemotherapy). The percentages of CR and nausea control in the acute, delayed, and overall phases were compared between the two treatment groups. Olanzapine toxicity was another outcome.

### Data Collection

A data collection form was designed to include patients’ demographic and clinical characteristics, diagnosis, chemotherapy regimen, risk of chemotherapy, prophylactic antiemetic regimens, and study outcomes. These data were extracted from patients’ files and through patient direct and telephone interviews.

### Patient Follow-Up and Assessment Procedure

To assess the study outcomes, patients were followed up by telephone interview daily from day 1 through 5. Patients were followed up during only one chemotherapy cycle. The interviewer asked the patient and recorded the study outcomes and any episodes of nausea or vomiting, the need for rescue antiemetic medications, and olanzapine toxicities (sedation or drowsiness and extrapyramidal symptoms). The interviewer assessed the severity of sedation, ranging from mild to moderate to severe, reflecting grade 1, grade 2, and grade 3 on Common Terminology Criteria of Adverse Effects. The interviewer also assessed whether the patient reduced the olanzapine dose to 5 mg/d or stopped olanzapine because of sedation or had life-threatening consequences (grade 4 toxicity) requiring urgent intervention.

### Statistical Analysis

Frequencies and percentages were obtained for each of the categorical variables. Mean and median were performed for continuous variables. The comparisons between the two groups were assessed using Pearson’s χ^2^. We performed subgroup analysis using crosstabs to evaluate the study outcomes of chemotherapy-naïve patients receiving HEC or MEC, chemotherapy-naïve patients receiving HEC, and chemotherapy-naïve patients receiving MEC. A two-tailed level of significance at *P* value of < .05 was considered significant and applied to all statistical tests. (SPSS v20; IBM, Armonk, NY) was used for all statistical analyses.

## RESULTS

### Patients' Demographic and Clinical Characteristics

**Fig 1:**
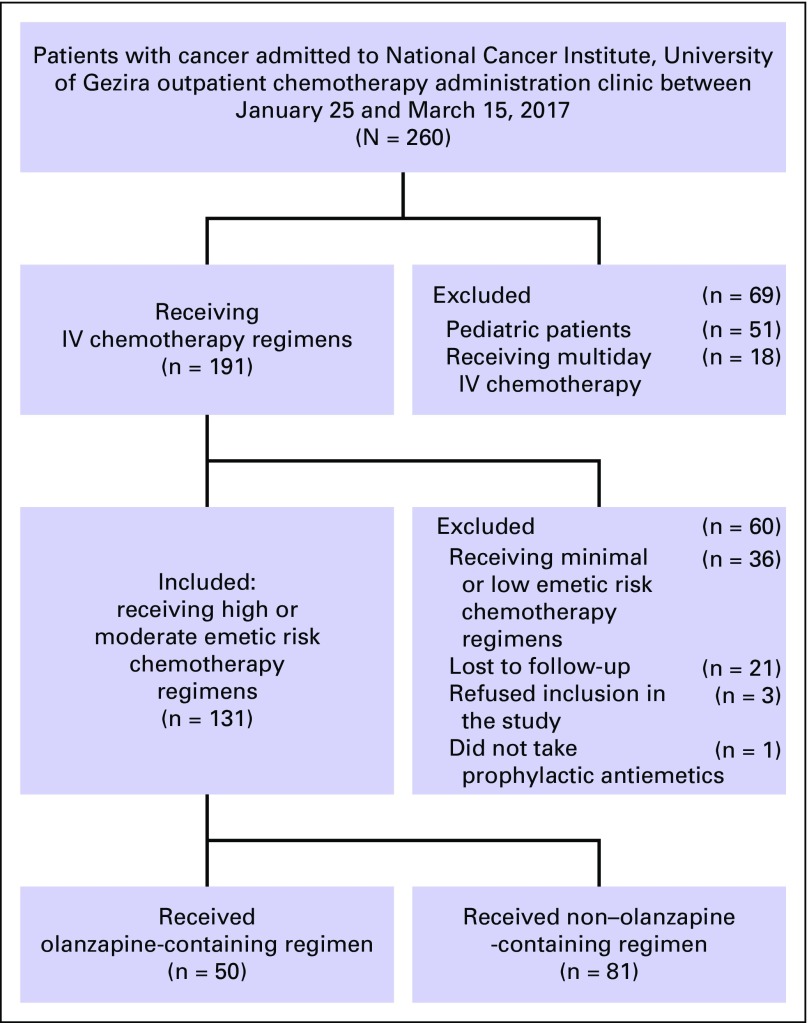
Study population. IV, intravenous.

Figure 1 shows distribution of our study population. A total of 131 patients (50.4%) out of 260 patients with cancer admitted to NCI-UG’s outpatient chemotherapy administration clinic during the study period met the inclusion criteria (olanzapine-containing regimen: 50 patients; non–olanzapine-containing regimen: 81 patients). Patients’ demographic and clinical characteristics are listed in Table 1. In both groups, the majority of patients were women and age ≤ 55 years. Breast cancer was the most frequent diagnosis, and the majority of patients received HEC regimens.

**Table 1:**
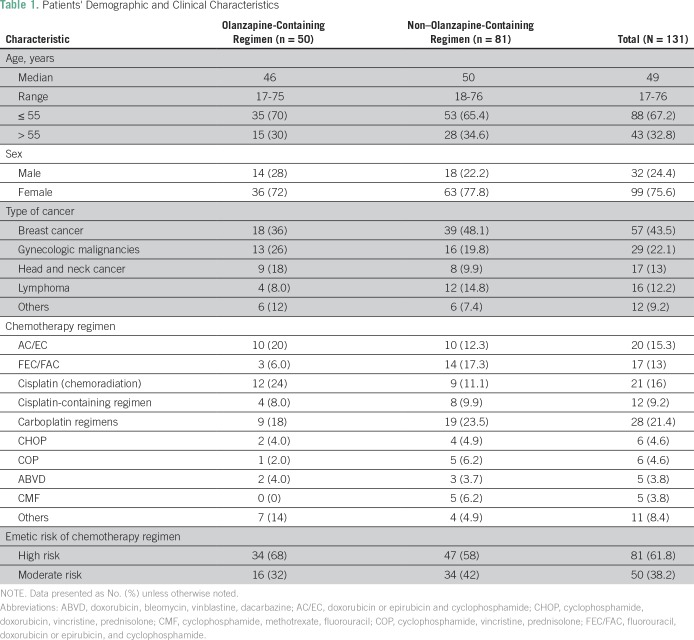
Patients' Demographic and Clinical Characteristics

### Outcomes of the Prophylactic Antiemetic Regimens

Table 2 lists higher percentages of patients achieved CR in the olanzapine-containing regimen compared with the non–olanzapine-containing regimen in the acute phase (86% *v* 71.6%; *P* = .086), delayed phase (72% *v* 30.9%; *P* < .001), and overall phase (66% *v* 25.9%; *P* < .001). Also, higher percentages of patients had complete nausea control in the olanzapine-containing regimen compared with the non–olanzapine-containing regimen in the acute phase (86% *v* 74.1%; *P* = .127), delayed phase (76% *v* 34.6%; *P* < .001), and overall phase (72% *v* 29.6%; *P* < .001). Table 3 lists the differences in outcomes between the two groups in different subgroup analysis.

**Table 2:**
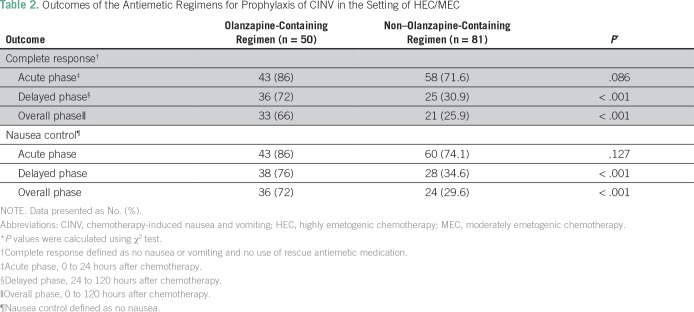
Outcomes of the Antiemetic Regimens for Prophylaxis of CINV in the Setting of HEC/MEC

**Table 3:**
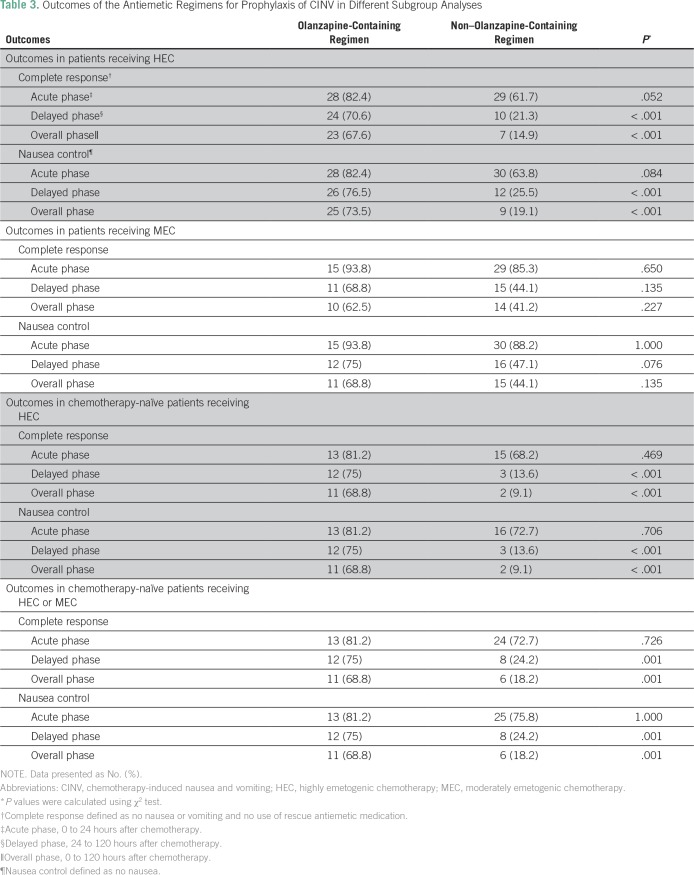
Outcomes of the Antiemetic Regimens for Prophylaxis of CINV in Different Subgroup Analyses

### Olanzapine Toxicity

Twenty-five patients had grade 1 or 2 sedation or drowsiness. Five patients had grade 3 sedation, and the olanzapine dose was reduced to 5 mg/d in four of them. None of our patients stopped olanzapine because of sedation or had life-threatening consequences (grade 4 toxicity) requiring urgent intervention. Thirteen patients had adverse effects not attributed to olanzapine (cough, constipation, fever, abdominal spasm, diarrhea, warmth, sweating, tachycardia, anorexia).

## DISCUSSION

CINV is an adverse event that should be totally controlled to improve quality of life and avoid complications. NK1RA-containing regimens are considered the standard of care for prophylaxis of CINV in patients receiving HEC or MEC, because they have shown higher efficacy in controlling CINV.^15^ Agents of these regimens, such as aprepitant and palonosetron, are expensive, and patients in developing countries cannot afford them.^16^

A previous observational study conducted at NCI-UG showed that the most commonly used prophylactic antiemetic regimen was a combination of ondansetron and dexamethasone (acute phase: ondansetron 8 to 16 mg IV and dexamethasone 8 to 16 mg IV; delayed phase: ondansetron 8 mg by mouth twice a day). None of patients receiving HEC or MEC received an NK1RA agent, because these agents are expensive and were not registered in Sudan, and this resulted in suboptimal control of CINV (CR in delayed phase, 36%).^19^ Therefore, to optimize prevention of CINV in our limited-resource setting, we need to adopt regional guidelines that take into account the economic feasibility of antiemetic agents.

Olanzapine is an antipsychotic agent that has the property of blocking multiple receptor types involved in acute and delayed emesis, including dopamine and serotonin receptors.^18^ Antiemetic regimens including olanzapine attained high efficacy in controlling CINV, and this was reflected in many clinical trials.^20-22^ A phase III clinical trial compared the efficacy of an olanzapine-containing regimen (olanzapine, palonosetron, and dexamethasone, OPD) versus an NK1RA-containing regimen (aprepitant, palonosetron, and dexamethasone, APD) for the prevention of CINV in patients receiving HEC regimens. In this trial, OPD was as effective as APD in emesis control in the acute (CR, 97% *v* 87%), delayed (CR, 77% *v* 73%), and overall phases (CR, 77% *v* 73%) and in acute nausea control (CR, 87% in both groups). Moreover, OPD was more effective in nausea control in delayed (CR, 69% *v* 38%) and overall phases (CR, 69% *v* 38%).^22^ On the basis of this trial, NCCN antiemetic guidelines added OPD as another less costly option for prevention of CINV in HEC and MEC regimens.^17^

In the United States, a tablet of 10 mg olanzapine is less expensive than aprepitant 125 or 80 mg (23.30 US$ *v* 647.50 US$). Also, the use of OPD leads to a significant cost reduction of approximately US$ 554 (APD, 1,143 US$; OPD, 589 US$).^16^ The introduction of a cost-saving alternative to NK1RA-containing regimens is a particular advantage for those working in resource-limited settings such as Sudan.

In this study, the olanzapine-containing regimen showed slightly higher percentages of CR and nausea control in the acute phase compared with the non–olanzapine-containing regimen (*P* > .05); this is because both arms contain ondansetron. Agents of the 5-HT3RA are well known to be effective in controlling acute emesis, because they block serotonin, which is an important mediator of acute emesis.^18^ In the delayed phase, the olanzapine-containing regimen was superior to the non–olanzapine-containing regimen in CR and nausea control (*P* < .05); this is again attributed to the use of ondansetron. 5-HT3RA agents are not effective in controlling delayed emesis, because delayed emesis occurs via different mechanisms that involve neurotransmitters other than serotonin. On the other hand, olanzapine has the property of blocking multiple receptor types involved in acute and delayed emesis, including dopamine and serotonin receptors.^18^

Our finding is consistent with a previous study, in which an olanzapine-containing regimen (olanzapine, azasetron, and dexamethasone) showed improved nausea control and vomiting control in the delayed and overall phases compared with the control group (azasetron and dexamethasone) in patients receiving HEC or MEC regimens (*P* < .05). In the acute phase, the percentages of nausea and vomiting control were slightly higher in the olanzapine-containing regimen (*P* > .05).^18^

The great effect of olanzapine-containing regimens led to other trials investigating the benefit of adding olanzapine to NK1RA-containing regimens. In these trials, the addition of olanzapine resulted in improved nausea and emesis control in the three phases.^5,23,24^ Recently the NCCN guideline version 2.2017 included olanzapine-NK1RA–containing regimens as a prophylactic option for patients receiving HEC regimens and experienced significant emesis in the previous cycle while using either olanzapine-containing or NK1RA-containing regimens^10^

In the current study, the major toxicity reported in patients who received the olanzapine-containing regimen was grade 1 and 2 sedation or drowsiness. A phase I clinical trial found that an olanzapine 10-mg dose was the maximum tolerated dose that resulted in emesis control with no dose-limiting toxicities.^25^ Since this trial, many subsequent clinical trials that evaluate olanzapine for prophylaxis of CINV use this 10-mg dose of olanzapine. In these trials, the major adverse effects of olanzapine were sedation or drowsiness,^1,24^ dizziness,^1^ and sleepiness.^18^ In these trials, olanzapine was not associated with grade 3 or 4 toxicities or with extrapyramidal symptoms, hyperglycemia, or weight gain, which are toxicities that are reported when olanzapine is used as an antipsychotic agent at higher doses and for longer period of time.^1,18,20,21^

To our knowledge, this study is the first in Sudan evaluating olanzapine for prevention of CINV. On the basis of our findings, the NCI-UG’s scientific meeting recommended the addition of olanzapine to the current antiemetic regimen for prevention of CINV in the setting of HEC/MEC.

The limitations of this study were its small size and the inclusion of non–chemotherapy-naïve patients, which may substantially affect the outcomes of the study. A well-designed RCT that includes only chemotherapy-naïve patients is recommended to improve the control of CINV in the setting of HEC/MEC.

This study demonstrated that an olanzapine-containing regimen, compared with a dexamethasone/ondansetron regimen, has better efficacy for prevention of delayed and overall emesis and nausea in patients receiving HEC or MEC regimens. Olanzapine also showed a better adverse effect profile. Oncologists working in a limited-resource setting should be familiar with olanzapine-containing regimens, because the NK1RA agents are not affordable and not easily available.
